# Late-week surgical treatment of endometrial cancer is associated with worse long-term outcome: Results from a prospective, multicenter study

**DOI:** 10.1371/journal.pone.0182223

**Published:** 2017-08-03

**Authors:** Tormund S. Njølstad, Henrica M. Werner, Janusz Marcickiewicz, Solveig Tingulstad, Anne C. Staff, Klaus Oddenes, Line Bjørge, Marie E. Engh, Kathrine Woie, Jostein Tjugum, Margaret S. Lode, Frederic Amant, Helga B. Salvesen, Jone Trovik

**Affiliations:** 1 Department of Clinical Science, University of Bergen, Haukeland University Hospital, Bergen, Norway; 2 Department of Obstetrics and Gynecology, Haukeland University Hospital, Bergen, Norway; 3 Centre for Cancer Biomarkers (CCBIO), University of Bergen, Bergen, Norway; 4 Department of Gynecology, Sahlgrenska Academy, University of Gothenburg, Gothenburg, Sweden; 5 Department of Obstetrics and Gynecology, Halland’s Hospital Varberg, Varberg, Sweden; 6 Department of Gynecology, St. Olav’s Hospital, Trondheim, Norway; 7 University of Oslo and Department of Gynecology, Oslo University Hospital, Ullevål, Oslo, Norway; 8 Department of Obstetrics and Gynecology, Haugesund Hospital, Haugesund, Norway; 9 Department of Gynecology, Akershus University Hospital, Lørenskog, Norway; 10 Department of Obstetrics and Gynecology, Førde Central Hospital, Førde, Norway; 11 Department of Obstetrics and Gynecology, Ålesund Hospital, Ålesund, Norway; 12 Gynecologic Oncology, University Hospitals Leuven, KU Leuven, Leuven, Belgium; 13 Center for Gynecologic Oncology (CGOA), Antoni van Leeuwenhoek, Netherlands Cancer Institute, Amsterdam, Netherlands; Tata Memorial Centre, INDIA

## Abstract

Surgery is the cornerstone in primary endometrial cancer treatment, and with curative intent it constitutes total hysterectomy and bilateral salpingo-oopherectomy. In addition, lymphadenectomy is performed in selected patients dependent on a preoperative risk assessment. Recent reports from the surgical approach to esophageal cancer reveal worse outcome when esophagectomy is performed later in the week. On this basis, we set out to explore weekday of surgery in relation to long-term outcome in 1302 endometrial cancer patients prospectively included in the MoMaTEC multicenter study. Day of surgery was dichotomized as early-week (Monday-Tuesday) or late-week (Wednesday-Friday), and evaluated as a discrete variable. Adjusted for patient age, Body Mass Index (BMI), FIGO stage, and histology, surgery performed later in the week was associated with 50.9% increased risk of all-cause death (p = 0.029). Among high-stage patients (FIGO stage III and IV), 5-year disease-specific survival proportions were 53.0% for early-week operated vs. 40.2% for late-week operated (p = 0.005 for difference). In multivariate survival analysis of high-stage patients, late-week surgery correlated with an increased risk of disease-specific death by 88.7% and all-cause death by 76.4% (p<0.017). Evaluating only patients who underwent lymphadenectomy, the adverse prognostic effect of being operated late-week remained for both disease-specific and all-cause death (HR 2.151 and HR 1.912, p = 0.004). Whether surgery was performed early- or late-week was not influenced by patient age, BMI, preoperative histology risk classification, FIGO stage or postoperative histology (all p>0.05). In conclusion, endometrial cancer surgery conducted late-week is associated with worse long-term outcome. Our findings are most evident among patients with higher FIGO stages, and patients who underwent more extensive surgical procedure (lymphadenectomy). With support from other studies, our results suggest that high-risk patients may benefit from surgery earlier in the week.

## Introduction

Endometrial cancer is the most common gynecological malignancy in the western world. The prognosis is generally favorable, with 5-year disease-specific survival reported to 80–90% [1, 2]. However, endometrial cancer is a heterogeneous disease with great variations in aggressiveness, and among patients with presumed localized disease recurrences are observed in 15–20% [3].

Surgery is the cornerstone in endometrial cancer treatment, and with curative intent constitutes total hysterectomy and bilateral salpingo-oophorectomy. In addition, retroperitoneal lymph node dissection is performed on a selection of patients dependent on a preoperative risk assessment based on tumor characteristics as well as patient operability. Long-term prognostic factors have been comprehensively investigated, the most important being the surgical FIGO stage (International Federation of Gynecology and Obstetrics), myometrial invasion, histological type, and differentiation grade [4].

Increased 30-day mortality in patients undergoing elective surgical procedures on Fridays compared to earlier in the week has been demonstrated in several large cohort studies [5, 6]. Long-term prognostic impact of surgery weekday is less explored. Recent reports from esophageal cancer have demonstrated increased 5-year mortality among patients with potentially curable disease when surgery is performed later in the week [7]. Furthermore, the prognostic effect was found to be independent of reoperations and short-term (30-day) mortality, leaving mechanisms involved unclear [8]. On this basis we set out to investigate long-term prognostic impacts of weekday of surgery for endometrial cancer patients undergoing primary surgical treatment.

## Materials and methods

### MoMaTEC study

After obtaining written informed consent, a total of 1372 patients from 10 centers were included in the MoMaTEC study (Molecular Markers in Treatment of Endometrial Cancer, Clinical Trial Identifier NCT00598845) between May 2001 and February 2013. The study obtained Institutional review board approval (REKIII no 052.01), and has acquired approval of the Norwegian Data Inspectorate (961478–2) and Norwegian Social Sciences Data services (15501). More comprehensive descriptions of the MoMaTEC study have been given elsewhere [9, 10].

Detailed clinicopathological data including age at diagnosis, date of primary surgical treatment, treatment modality, tumor FIGO stage (2009 criteria), and histology from hysterectomy specimens were recorded. Histological type and grade were categorized as endometrioid grade 1 or 2, endometrioid grade 3, or non-endometrioid histology. Preoperative curettage histology reports were routinely categorized as high-risk (non-endometrioid, and histological grade 3 endometrioid carcinomas) or low-risk (endometrioid carcinoma grade 1 or 2, hyperplasia and benign endometrium). Participating centers applied local adjuvant treatment protocols, individually tailored for each patient by the responsible physician. Follow-up data were collected from patient records and correspondence with the respective physicians responsible for outpatient controls.

### Weekday of surgery and statistical analysis

For this study, the weekday of performed surgical procedure was obtained from recorded date of primary surgery in a post hoc fashion. When exploring categorical associations between variables and univariate survival analyses, day of surgery was dichotomized as either early-week (Monday or Tuesday) or late-week (Wednesday through Friday), in concordance with the study on esophageal cancer patients [7]. For multivariate survival analysis, weekday of surgery was explored both categorical and as a discrete variable to evaluate for linear trends. For the latter, the following coding was applied: 1 = Monday, 2 = Tuesday, 3 = Wednesday, 4 = Thursday, 5 = Friday.

Statistical analyses were performed by SPSS, Statistical Package for the Social Sciences, version 22.0 (IBM, Chicago, IL, USA). Associations between categorical variables were investigated using the Pearson’s χ^2^-test. For comparisons of mean and median, a Student’s t-test and Mann-Whitney U-test were applied as appropriate. Univariate survival analyses were conducted by the Kaplan-Meier method with log-rank test for statistical significance, and multivariate survival analysis by the Cox proportional hazards model for Hazard Ratios (HR). All tests were two-sided, and considered statistically significant when a probability of less than 0.05 was observed.

## Results

### Demographic, clinicopathological and treatment characteristics

Of the 1372 MoMaTEC patients treated for primary endometrial cancer by hysterectomy and bilateral salpingo-oopherectomy, 1302 patients were included in the final analyses. Six patients were discarded for being treated on a Sunday, and 64 patients were discarded due to lack of follow-up information.

Patient age ranged from 25 to 98 years, with a mean of 65.9 years. Mean follow-up time was 35.8 months (range 0 to 105 months). Details of demographic, surgical and clinicopathological characteristics are presented in Table 1, along with the corresponding distribution to weekday of performed surgical procedure.

**Table 1:** Clinicopathological characteristics and demographics of endometrial cancer patients treated in the MoMaTEC* study.

Characteristics	Total Cohort		Weekday of Surgery				p-value^a^
			Monday-Tuesday		Wednesday-Friday		
	Number, %		Number, %		Number, %		
Total	1302	100%	723	100%	579	100%	
**Preoperative characteristics**							
*Age*							
<66 years	643	49.4%	365	50.5%	278	48.0%	
≥ 66 years	659	50.6%	358	49.5%	301	52.0%	0.376^b^
*Body Mass Index (BMI)*							
<25 kg/m^2^	229	35.1%	123	36.9%	106	33.2%	
≥25 kg/m^2^	423	64.9%	210	63.1%	213	66.8%	0.321^c^
*Curettage histology classification*							
Low-risk	984	77.7%	548	77.4%	436	78.1%	
High-risk	282	22.3%	160	22.6%	122	21.9%	0.775
**Treatment details**							
Performed *lymphadenectomy*							
Yes	977	75.0%	521	72.1%	456	78.8%	
No	325	25.0%	202	27.9%	123	21.2%	0.006
*Adjuvant treatment*^d^							
No treatment	913	70.1%	512	70.8%	401	69.3%	
Adjuvant treatment	389	29.9%	211	29.2%	178	30.7%	0.541
**Postoperative characteristics**							
*FIGO stage*^e^							
Stage I / II	1114	85.6%	626	86.6%	488	84.3%	
Stage III / IV	188	14.4%	97	13.4%	91	15.7%	0.241
*Histological subtype and grade*							
Endometrioid grade 1–2	898	69.5%	503	70.2%	395	68.6%	
Endometrioid grade 3	156	12.1%	80	11.2%	76	13.2%	
Non-endometrioid	239	18.5%	134	18.7%	105	18.2%	0.535
*Myometrial infiltration*							
<50%	802	66.4%	454	68.3%	348	64.2%	
≥50%	405	33.6%	211	31.7%	194	35.8%	0.137
*Lymph node metastasis*^f^							
No	858	87.7%	454	87.1%	404	88.4%	
Yes	120	12.3%	67	12.9%	53	11.6%	0.548

Clinicopathological characteristics and demographics related to weekday of surgery for 1302 endometrial cancer patients undergoing primary surgical treatment. Weekday of surgery grouped as early-week (Monday and Tuesday) or late-week (Wednesday through Friday).

*MoMaTEC: Molecular Markers in Treatment of Endometrial cancer

^a^Pearson χ^2^ test for two-sided significance.

^b^p = 0.131 when testing age as a continuous variable by Student’s t-test for equality of means.

^c^p = 0.779 when testing BMI as a continuous variable by Mann-Whitney U test for equality of medians.

^d^Adjuvant treatment comprising external radiation, internal radiation, chemotherapy, chemoradiation or hormonal treatment.

^e^FIGO = International Federation of Gynecology and Obstetrics (according to 2009 revision)

^f^Lymph node metastasis evaluated among 977 patients with performed lymphadenectomy

Most patients (96.1%) were treated Monday through Thursday (n = 1251). In detail, 26.8% were treated on a Monday (n = 349), 28.7% on a Tuesday (n = 374), 22.0% on a Wednesday (n = 286), 18.6% on a Thursday (n = 242) and 3.9% on a Friday (n = 51).

Lymphadenectomy was performed in 75.0% of cases (n = 977). Adjuvant treatment was given to 29.9% of patients (n = 389) as various combinations of chemotherapy (n = 246) and/or radiotherapy (n = 195), or hormonal treatment (n = 5). As portrayed in Table 1, comparing patients with performed surgery early-week to late-week, no significant difference was observed in regard to patient age, BMI, preoperative curettage risk classification, FIGO stage, hysterectomy specimen histology (type and grade), myometrial infiltration or adjuvant treatment (all p>0.05). An overview of specific adjuvant treatment received in relation to weekday of primary surgery is presented for all patients, high-stage patients, and patients with performed lymphadenectomy as supplementary material (S1 Table). We observed a tendency towards higher rate of lymph node sampling late-week (78.8% of patients) compared with early-week surgery (72.1% of patients) (p = 0.006). Furthermore, the number of nodes removed was significantly higher when operated early-week (a mean of 16.9 removed nodes) compared to late-week (a mean of 15.3 nodes, p = 0.022 for difference). However, there was no significant difference in the presence of lymph node metastasis between early- or late-week surgeries on postoperative histological scrutiny (p = 0.557).

### Univariate survival analysis

**Fig 1:**
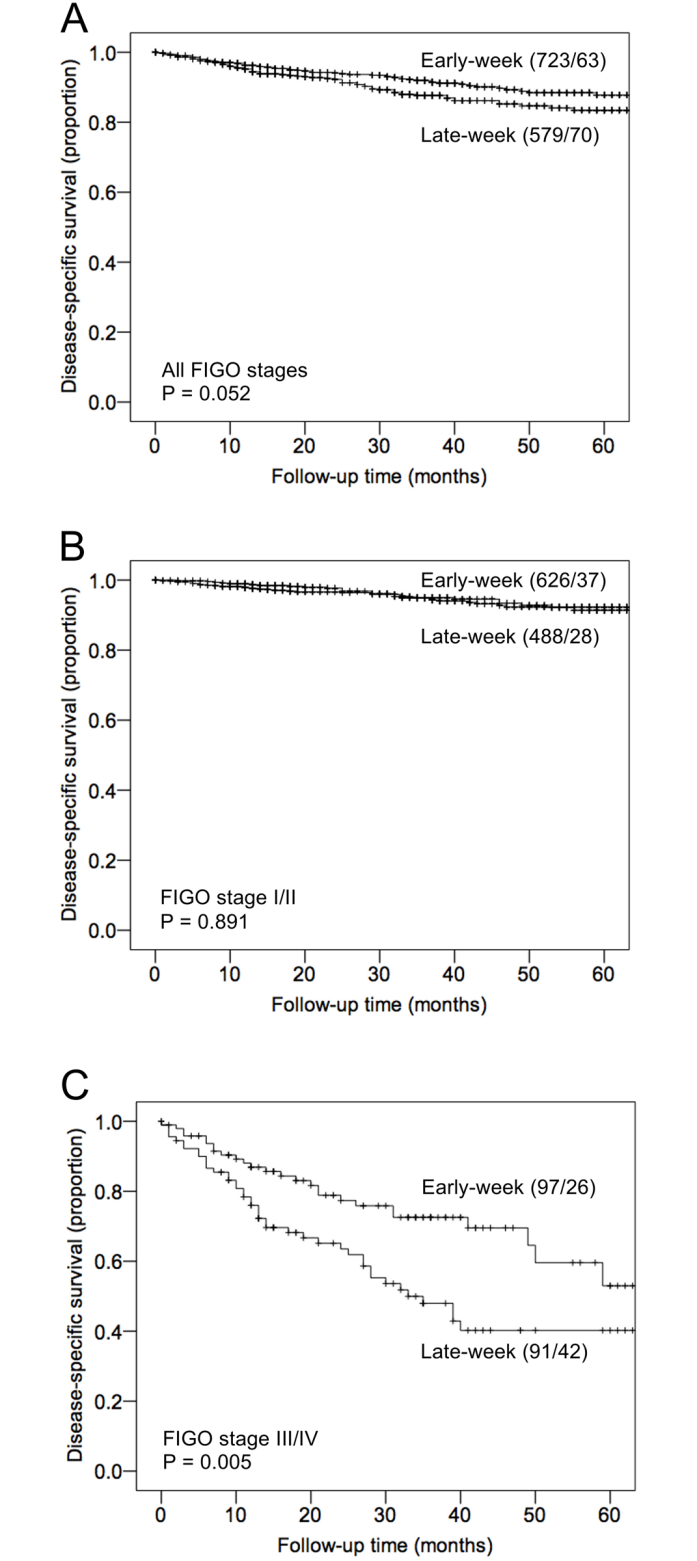
Disease-specific survival proportions in relation to weekday of surgery. Weekday of surgery categorized as early-week (Monday and Tuesday) or late-week (Wednesday to Friday). A) All patients (n = 1302), B) patients with low FIGO stages (n = 1114), and C) patients with high FIGO stages (n = 188). For each category, the number of cases followed by the number of deaths is given in parenthesis. P-values are by the Kaplan-Meier estimation by the log-rank test.

Survival curves for univariate disease-specific survival proportions (DSS) are presented in Fig 1, and for overall survival as supplementary material (S1 Fig). For all patients, surgery performed early-week was associated with borderline significant improved 5-year DSS proportions compared to patients operated late-week (87.8% vs. 83.4%, respectively; p = 0.052) (Fig 1A). A difference was also observed for overall survival, however not statistically significant (81.2% compared to 76.0%, p = 0.126) (S1A Fig).

To further explore the prognostic effects of weekday of surgery, subgroup analyses of low FIGO stages (stage I and II patients) and high FIGO stages (stage III and IV patients) were performed separately. Among patients with low FIGO stages, there was no statistical significant difference in survival between early- and late-week operated for either DSS (Fig 1B) or overall survival (S1B Fig). However, evaluating patients with high FIGO stages, a significant difference in survival was observed. Five-year DSS proportions were 53.0% among early-week operated, compared to 40.2% among late-week operated (p = 0.005, Fig 1C), and overall survival proportions were 48.1% among early-week operated compared to 37.2% among late-week operated (p = 0.004, S1C Fig). An overview of 5-year survival proportions according to each weekday of performed surgery is presented as supplementary material (S2 Table).

### Multivariate survival analysis

Results from multivariate Cox regression analyses are listed in S3 Table. Adjusted for patient age, BMI, FIGO stage and histology, being operated late-week was associated with an increased risk of all-cause death estimated to 50.9% (HR 1.509, 95% CI 1.042–2.186, p = 0.029). For disease-specific death, the increased risk was estimated to 52.1%, however of borderline significance (HR 1.521, 95% CI 0.988–2.342, p = 0.057).

Subgroup analyses for patients with high FIGO stages (stage III and IV) are presented in Table 2. Corrected for patient age and tumor histology, late-week surgery was associated with an increased risk of disease-related death of 88.7% (HR 1.887, 95% CI 1.146–3.108, p = 0.013) and all-cause death of 76.4% (HR 1.764, 95% CI 1.105–2.816, p = 0.017) (Table 2). Including BMI as a covariate reduced the number of patients with complete data, but did not change the significance of our results.

**Table 2:** Multivariate survival analyses of high stage endometrial cancer patients with weekday of surgery as a grouped variable.

		Disease-specific survival			Overall survival		
Variable	n	Adjusted HR	95% CI	P	Adjusted HR	95% CI	P
*Age at primary treatment*^a^	187	1.037	1.014–1.061	0.002	1.047	1.024–1.070	<0.001
*Histology*^b^							
Endometrioid grade 1–2	76	1	-	-	1	-	-
Endometrioid grade 3	38	1.915	0.984–3.728	0.056	1.569	0.834–2.952	0.162
Non-endometrioid	73	1.918	1.082–3.400	0.026	1.725	1.018–2.923	0.043
*Weekday of surgery*							
Monday-Tuesday	97	1	-	-	1	-	-
Wednesday-Friday	90	1.887	1.146–3.108	0.013	1.764	1.105–2.816	0.017

Multivariate disease-specific and overall survival analyses according to the Cox proportional hazards regression model for 187 endometrial cancer patients with high FIGO stages (stage III-IV). Weekday of surgery was dichotomized as either early-week (Monday and Tuesday), or late-week (Wednesday through Friday).

CI = Confidence Interval, FIGO = International Federation of Gynecology and Obstetrics, HR = Hazard Ratio.

^a^Age of primary treatment evaluated as a continuous variable.

^b^Histology evaluated in postoperatively acquired hysterectomy specimens

Multivariate survival analyses of high-stage patients with weekday of surgery as a discrete variable are presented in Table 3. Corrected for age and tumor histology, an increment in weekday of surgery with Monday as the reference day increased risk of disease-specific death with an estimated 24.2% per day (HR 1.242, 95% CI 1.012–1.524, p = 0.038). The observed increased risk was slightly smaller when analyzing death from any cause (HR 1.197, 95% CI 0.986–1.453, p = 0.069).

**Table 3:** Multivariate survival analyses of high stage endometrial cancer patients with weekday of surgery as a discrete variable.

		Disease-specific survival			Overall survival		
Variable	n	Adjusted HR	95% CI	p-value	Adjusted HR	95% CI	p-value
*Age at primary treatment*^a^	187	1.039	1.016–1.063	0.001	1.049	1.026–1.072	<0.001
*Histology*^b^							
Endometrioid grade 1–2	76	1	-	-	1	-	-
Endometrioid grade 3	38	1.884	0.969–3.662	0.062	1.550	0.826–2.910	0.173
Non-endometrioid	73	1.898	1.072–3.362	0.028	1.709	1.009–2.895	0.046
*Weekday of surgery*^c^	187	1.242	1.012–1.524	0.038	1.197	0.986–1.453	0.069

Multivariate disease-specific and overall survival analyses according to the Cox proportional hazards regression model for 187 endometrial cancer patients with high FIGO stages (FIGO stage III-IV). Weekday of surgery is evaluated as a discrete variable.

CI = Confidence Interval, FIGO = International Federation of Gynecology and Obstetrics, HR = Hazard Ratio.

^a^Age of primary treatment evaluated as a continuous variable.

^b^Histology evaluated in postoperatively acquired hysterectomy specimens

^c^Weekday of surgery evaluated as a discrete variable encoded 1 = Monday, 2 = Tuesday, 3 = Wednesday, 4 = Thursday, 5 = Friday

Assessing only patients with performed lymphadenectomy, late-week surgery remained an independent prognostic factor (Table 4). Corrected for age, BMI, FIGO stage and histology, late-week surgery associated with a significant increased risk of disease-specific death (HR 2.151, 95% CI 1.280–3.614, p = 0.004) and all-cause death (HR 1.912, 95% CI 1.232–2.968, p = 0.004). Evaluating weekday of surgery as a discrete variable, the increased risk per incremental weekday was estimated to 25.0% for disease-related death (HR 1.250, 95% CI 1.017–1.537, p = 0.034), and 22.5% for all-cause death (HR 1.225, 95% CI 1.024–1.466, p = 0.026) with Monday as the reference category.

**Table 4:** Multivariate survival analyses of all patients with performed lymphadenectomy.

		Disease-specific survival			Overall survival		
Variable	n	Adjusted HR	95% CI	p-value	Adjusted HR	95% CI	p-value
*Age at primary treatment*^a^	529	1.044	1.016–1.072	0.002	1.053	1.029–1.077	<0.001
*BMI*^a^	529	0.982	0.936–1.030	0.453	1.000	0.961–1.040	0.982
*FIGO stage (2009)*							
Stage I/II	443	1	-	-	1	-	-
Stage III/IV	86	6.545	3.809–11.245	<0.001	4.497	2.824–7.160	<0.001
*Histology*^b^							
Endometrioid grade 1–2	340	1	-	-	1	-	-
Endometrioid grade 3	75	4.429	1.925–10.188	<0.001	1.972	1.012–3.843	0.046
Non-endometrioid	114	6.991	3.419–14.298	<0.001	3.545	2.065–5.779	<0.001
*Weekday of surgery*							
Monday-Tuesday	264	1	-	-	1	-	-
Wednesday-Friday	265	2.151	1.280–3.614	0.004	1.912	1.232–2.968	0.004

Multivariate disease-specific and overall survival analyses according to the Cox proportional hazards regression model for 529 endometrial cancer patients with performed lymphadenectomy. Weekday of surgery was dichotomized as either early-week (Monday and Tuesday), or late-week (Wednesday through Friday).

CI = Confidence Interval, FIGO = International Federation of Gynecology and Obstetrics, HR = Hazard Ratio.

^a^Age of primary treatment and BMI evaluated as continuous variables.

^b^Histology evaluated in postoperatively acquired hysterectomy specimens

High-volume centers (n = 4) contributed the majority of patients (n = 1116) while low-volume centers (n = 6) each contributed <100 patients each. When comparing centers, we observed that low-volume centers had a tendency of performing surgery earlier in the week, with 71.5% of patients operated early-week versus 52.9% for high-volume centers (p<0.001 for difference). All analyses were therefore applied to patients from high-volume centers only, to avoid this potential confounder. However, late-week surgery remained associated with an increased risk of all-cause death (HR 1.533, 95% CI 1.056–2.224, p = 0.025) as well as disease-specific death (HR 1.554, 95% CI 1.006–2.401, p = 0.047), corrected for FIGO stage, histology, patient age and BMI. Evaluating patients of high-stage only from high-volume centers, the significance of our results was not altered.

## Discussion

Our results indicate that endometrial cancer surgery performed later in the week is associated with worse long-term outcome. The prognostic effect is more evident among patients with higher tumor stages, as well as patients with more extensive surgery performed (lymphadenectomy). Interestingly, our observed prognostic effects apply to both disease-specific as well as all-cause death. With support from other studies, this suggests that complex patients with advanced tumor stages and planned extensive surgery may benefit from being operated earlier in the week.

Recently, a large population-based cohort study by Lagergren *et al* [7] evaluating surgical resection of esophageal cancer found improved long-term outcome for patients operated early-week compared to late-week. Noticeably, their observed prognostic impact was more evident for patients with lower tumor stages. By comparison, our study finds the prognostic effect to be more evident among patients with higher tumor stages, and one may in this regard hypothesize that the surgical skills essential for long-term survival are most crucial in advanced stage endometrial cancer, and low-stage esophageal cancer. Furthermore, while esophageal cancer surgery may be regarded as extensive (Lagergren *et al* report an average operating time for esophagectomy of 6 ½ hours), endometrial cancer surgery requires approximately a third of that operating time and is associated with low procedure-related mortality. Our findings, demonstrated on a less extensive surgical procedure, are in this regard an interesting supplement to the matter of surgery weekday and long-term outcome.

A “weekend” effect of increased mortality for surgical procedures performed during the weekend is well described in several studies [11–13]. However, a “weekday” effect is less explored. Large cohort studies have demonstrated increased short-term (30-day) mortality in patients undergoing surgery on a Friday compared to earlier in the week [5, 6]. The detected increased short-term mortality has been attributed to reduction in clinical personnel [13, 14], lack of seniority and experience [15], poorer availability of services later in the week [16], and higher prevalence of complications within 48 hours of surgery [17]. However, it is also suggested that the effect may largely be attributed to an unmeasured severity of illness rather than differences in quality of care [18]. Contrasting these studies, our study demonstrates a long-term prognostic effect, suggesting that the effects of weekday of surgery may last beyond the immediate postoperative period. The cause is unknown. Interestingly, the follow-up study on esophageal cancer by *Lagergren et al* found no association between weekday of surgery and risk of reoperation, or increased 30-day mortality, suggesting other mechanisms affecting long-term prognosis [8]. One may speculate that a combination of the above-mentioned factors in regard to patient care, as well as possible deterioration of individual surgical precision towards the end of the week, come into play. However, it is also likely that unknown confounding variables account for the observed differences in survival between early- and late-week operated.

Our study is not without limitations. Comorbid conditions are not accounted for in detail in our population based prospective study design. However, by correcting for patient age and BMI as surrogate markers for comorbidity, and by investigating both disease-specific as well as all-cause mortality in our multivariate survival analyses, we have assumed to reduce the effect of this potential confounder. In addition, although our surgical complication rates and readmission rates are not known, biases related to unfavorable non-endometrial cancer related events have been adjusted for by investigating disease-specific survival. Furthermore, the quality of the follow-up in our multicenter study has an advantage over register-based studies, with detailed patient follow-up data from the individually responsible physician. Although our total sample size may be regarded as large (n = 1302), our observed differences in survival were strongest for a smaller subset of patients with high stage (n = 188). This calls for further support from other studies, and need for replication of our findings in larger sample sizes.

As illustrated in S2 Table, survival proportions seem to be better for patients operated Fridays compared to the days before. This conflicts with our postulated decreased survival towards end-of-week. However, we attribute this to be due to a small number of patients operated on Fridays (n = 51 of a total of 1302 patients), which is further supported by the standard error of the survival estimate for patients operated on Fridays being comparably large (S2 Table). Furthermore, as portrayed in Table 3, multivariate survival analyses with weekday of surgery as a discrete variable (including Friday) showed a significant increased risk of disease-specific death towards end-of-week.

We found no selection bias of patients to early- or late-week surgery depending on age, BMI, preoperative curettage histology, FIGO stage, presence of lymph node metastasis, or adjuvant treatment. As noted, we observed a collective trend of low-volume centers performing surgery earlier in the week. This is unlikely to have affected our results, as centers with lower rates of patient contribution constituted a minority of total included patients (n = 186 of 1302), and as our prognostic findings were confirmed in subgroup analysis of high-volume centers only.

In conclusion, our large population-based prospective multicenter study indicates that endometrial cancer surgery conducted later in the week is associated with worse long-term outcome. This effect was more evident among patients with higher tumor stages and more extensive surgery performed (lymphadenectomy). With support from other patient cohorts, this suggests that patients with advanced tumor stages and patients with planned extensive surgery may benefit from being operated early-week.

## Supporting information

S1 Fig Overall survival of endometrial cancer patients in relation to weekday of surgery, categorized as early-week (Monday and Tuesday) or late-week (Wednesday to Friday). A) All patients (n = 1302), B) patients with low FIGO stages (n = 1114), and C) patients with high FIGO stage (n = 188). For each category, the number of cases followed by the number of deaths is given in parenthesis. P-values are by the Kaplan-Meier estimation by the log-rank test.
(TIF)

S1 Table Specification of adjuvant treatment. Specification of adjuvant treatment grouped according to weekday of primary surgical treatment, for all patients (n = 1302), high-stage patients (n = 188) and patients with performed lymphadenectomy (n = 977).
^a^Pearson χ^2^ test for two-sided significance.
(DOCX)

S2 Table Weekday of surgery in relation to 5-year survival. Weekday of surgery in relation to 5-year survival proportions for 1302 endometrial cancer patients.
(DOCX)

S3 Table Multivariate survival analyses. Multivariate disease-specific and overall survival analyses according to the Cox proportional hazards regression model for 645 patients with endometrial cancer. Weekday of surgery was dichotomized as either early-week (Monday and Tuesday), or late-week (Wednesday through Friday).
CI = Confidence Interval, FIGO = International Federation of Gynecology and Obstetrics, HR = Hazard Ratio.
^1^Age of primary treatment and BMI evaluated as continuous variables.
^2^Histology evaluated in postoperatively acquired hysterectomy specimens.
(DOCX)
