# Supplementary figures and images for: Late-week surgical treatment of endometrial cancer is associated with worse long-term outcome: Results from a prospective, multicenter study
Source: PLoS One. 2017 Aug 3;12(8):e0182223. doi: 10.1371/journal.pone.0182223 (PMC5542466; doi:10.1371/journal.pone.0182223)

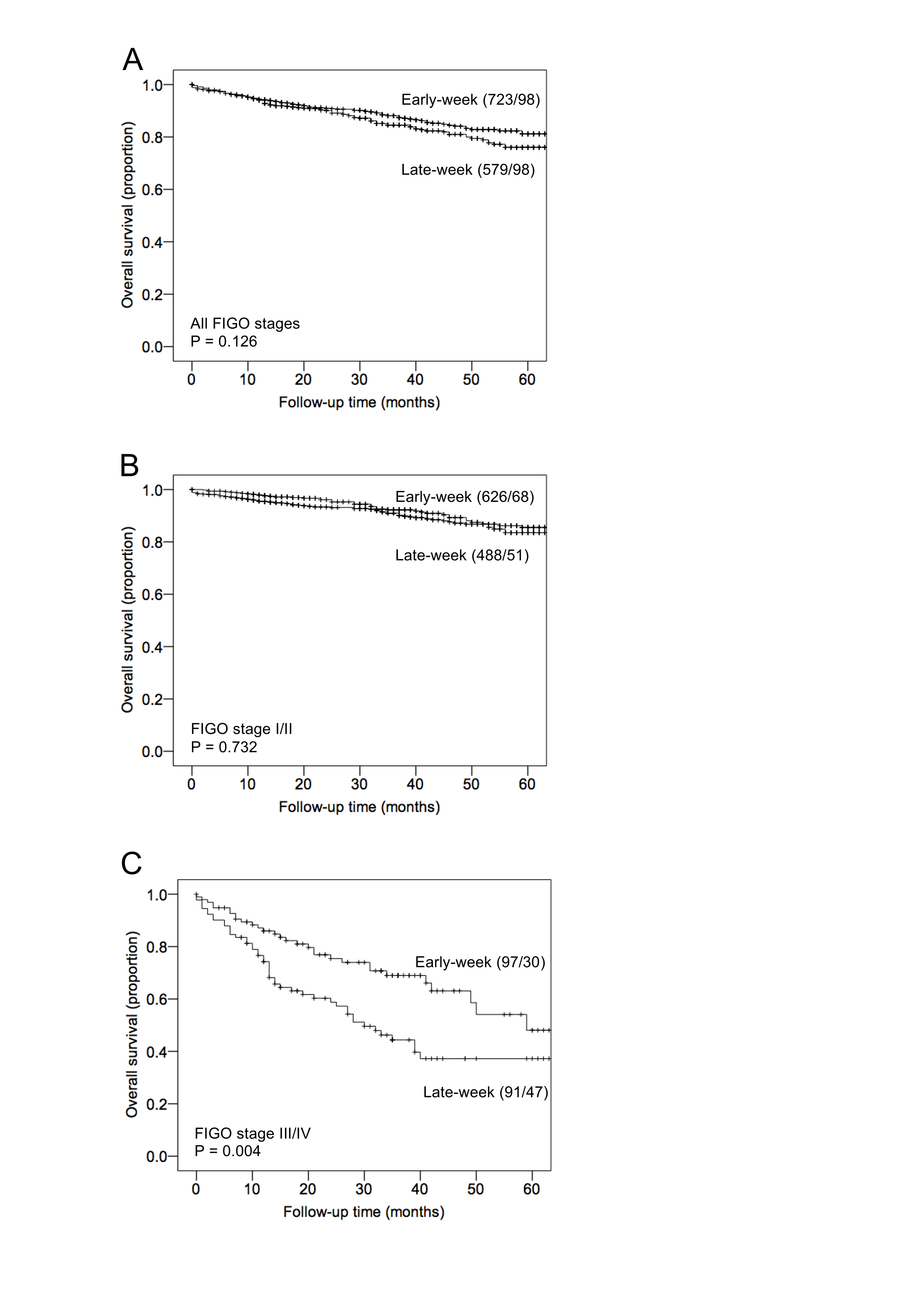

Supplement: S1 Fig — A) All patients (n = 1302), B) patients with low FIGO stages (n = 1114), and C) patients with high FIGO stage (n = 188). For each category, the number of cases followed by the number of deaths is given in parenthesis. P-values are by the Kaplan-Meier estimation by the log-rank test. (TIF) [file pone.0182223.s001.tif]
