# Supplementary material for: Late-week surgical treatment of endometrial cancer is associated with worse long-term outcome: Results from a prospective, multicenter study
Source: PLoS One. 2017 Aug 3;12(8):e0182223. doi: 10.1371/journal.pone.0182223 (PMC5542466; doi:10.1371/journal.pone.0182223)
Supplement: S1 Table — Specification of adjuvant treatment grouped according to weekday of primary surgical treatment, for all patients (n = 1302), high-stage patients (n = 188) and patients with performed lymphadenectomy (n = 977). aPearson χ2 test for two-sided significance. (DOCX) [file pone.0182223.s002.docx]

**S1 Table. Specification of adjuvant treatment.**

|  |  |  | | Weekday of Surgery | | | |  |
| --- | --- | --- | --- | --- | --- | --- | --- | --- |
|  | Total Cohort | | | Monday-Tuesday | | Wednesday-Friday | |  |
| All patients (n=1302) | Number, % | | | Number, % | | Number, % | | p-value^a^ |
| No adjuvant treatment | 913 | | 70.1% | 512 | 70.8% | 401 | 69.3% |  |
| External radiation | 85 | | 6.5% | 45 | 6.2% | 40 | 6.9% |  |
| Internal radiation | 53 | | 4.1% | 25 | 3.5% | 28 | 4.8% |  |
| Chemotherapy | 189 | | 14.5% | 108 | 14.9% | 81 | 14.0% |  |
| Hormonal treatment | 5 | | 0.4% | 2 | 0.3% | 3 | 0.5% |  |
| Chemoradiation | 57 | | 4.4% | 31 | 4.3% | 26 | 4.5% | 0.767 |
| Total | 1302 | | 100% | 723 | 100% | 579 | 100% |  |
|  |  | |  |  |  |  |  |  |
| High-stage patients (n=188) |  | |  |  |  |  |  |  |
| No adjuvant treatment | 24 | | 12.8% | 9 | 9.3% | 15 | 16.5% |  |
| External radiation | 38 | | 20.2% | 19 | 19.6% | 19 | 20.9% |  |
| Internal radiation | 4 | | 2.1% | 1 | 1.0% | 3 | 3.3% |  |
| Chemotherapy | 83 | | 44.1% | 47 | 48.5% | 36 | 39.6% |  |
| Hormonal treatment | 4 | | 2.1% | 1 | 1.0% | 3 | 3.3% |  |
| Chemoradiation | 35 | | 18.6% | 20 | 20.6% | 15 | 16.5% | 0.359 |
| Total | 188 | | 100% | 97 | 100% | 91 | 100% |  |
|  |  | |  |  |  |  |  |  |
| Patients with performed lymphadenectomy (n=977) |  | |  |  |  |  |  |  |
| No adjuvant treatment | 680 | | 69.6% | 360 | 69.1% | 320 | 70.2% |  |
| External radiation | 68 | | 7.0% | 37 | 7.1% | 31 | 6.8% |  |
| Internal radiation | 23 | | 2.4% | 8 | 1.5% | 15 | 3.3% |  |
| Chemotherapy | 168 | | 17.2% | 93 | 17.9% | 75 | 16.4% |  |
| Hormonal treatment | 3 | | 0.3% | 2 | 0.4% | 1 | 0.2% |  |
| Chemoradiation | 35 | | 3.6% | 21 | 4.0% | 14 | 3.1% | 0.498 |
| Total | 977 | | 100% | 521 | 100% | 456 | 100% |  |
|  |  | |  |  |  |  |  |  |

Specification of adjuvant treatment grouped according to weekday of primary surgical treatment, for all patients (n=1302), high-stage patients (n=188) and patients with performed lymphadenectomy (n=977).

^a^Pearson χ^2^ test for two-sided significance.
