# Supplementary material for: Late-week surgical treatment of endometrial cancer is associated with worse long-term outcome: Results from a prospective, multicenter study
Source: PLoS One. 2017 Aug 3;12(8):e0182223. doi: 10.1371/journal.pone.0182223 (PMC5542466; doi:10.1371/journal.pone.0182223)
Supplement: S2 Table — Weekday of surgery in relation to 5-year survival proportions for 1302 endometrial cancer patients. (DOCX) [file pone.0182223.s003.docx]

**S2 Table. Weekday of surgery in relation to 5-year survival**.

|  |  | | 5-year disease-specific survival | | 5-year overall survival | |
| --- | --- | --- | --- | --- | --- | --- |
| Weekday | Number, % | | Proportion, SE | | Proportion, SE | |
| Monday | 349 | 26.8% | 88.3% | 0.024 | 79.3% | 0.030 |
| Tuesday | 374 | 28.7% | 87.3% | 0.024 | 82.9% | 0.028 |
| Wednesday | 286 | 22.0% | 84.3% | 0.026 | 77.1% | 0.032 |
| Thursday | 242 | 18.6% | 82.1% | 0.033 | 74.0% | 0.040 |
| Friday | 51 | 3.9% | 86.2% | 0.077 | 84.3% | 0.077 |
| Monday-Tuesday | 725 | 55.7% | 87.8% | 0.017 | 81.2% | 0.020 |
| Wednesday-Friday | 577 | 44.3% | 83.4% | 0.020 | 76.0% | 0.025 |
| Total | 1302 | 100% | 85.8% | 0.013 | 78.9% | 0.016 |

Weekday of surgery in relation to 5-year survival proportion for 1302 endometrial cancer patients.

SE: Standard Error. MoMaTEC: Molecular Markers in Treatment of Endometrial Cancer
