# Supplementary material for: Late-week surgical treatment of endometrial cancer is associated with worse long-term outcome: Results from a prospective, multicenter study
Source: PLoS One. 2017 Aug 3;12(8):e0182223. doi: 10.1371/journal.pone.0182223 (PMC5542466; doi:10.1371/journal.pone.0182223)
Supplement: S3 Table — Multivariate disease-specific and overall survival analyses according to the Cox proportional hazards regression model for 645 patients with endometrial cancer. Weekday of surgery was dichotomized as either early-week (Monday and Tuesday), or late-week (Wednesday through Friday). CI = Confidence Interval, FIGO = International Federation of Gynecology and Obstetrics, HR = Hazard Ratio. 1Age of primary treatment and BMI evaluated as continuous variables. 2Histology evaluated in postoperatively acquired hysterectomy specimens. (DOCX) [file pone.0182223.s004.docx]

**S3 Table. Multivariate survival analyses.**

|  |  | Disease-specific survival | | | Overall survival | | |
| --- | --- | --- | --- | --- | --- | --- | --- |
| Variable | n | Adjusted HR | 95% CI | P | Adjusted HR | 95% CI | P |
| *Age at treatment^1^* | 645 | 1.041 | 1.020-1.064 | <0.001 | 1.052 | 1.033-1.072 | <0.001 |
|  |  |  |  |  |  |  |  |
| *BMI^1^* | 645 | 1.007 | 0.974-1.041 | 0.667 | 1.018 | 0.990-1.046 | 0.221 |
|  |  |  |  |  |  |  |  |
| *FIGO stage (2009)* |  |  |  |  |  |  |  |
| Stage I/II | 535 | 1 | - | - | 1 | - | - |
| Stage III/IV | 110 | 5.561 | 3.544-8.726 | <0.001 | 4.344 | 2.937-6.424 | <0.001 |
|  |  |  |  |  |  |  |  |
| *Histology^2^* |  |  |  |  |  |  |  |
| Endometrioid grade 1-2 | 422 | 1 | - | - | 1 | - | - |
| Endometrioid grade 3 | 91 | 2.879 | 1.526-5.431 | 0.001 | 1.845 | 1.068-3.188 | 0.028 |
| Non-endometrioid | 132 | 4.469 | 2.601-7.677 | <0.001 | 3.296 | 2.134-5.090 | <0.001 |
|  |  |  |  |  |  |  |  |
| *Weekday of surgery* |  |  |  |  |  |  |  |
| Monday-Tuesday | 329 | 1 | - | - | 1 | - | - |
| Wednesday-Friday | 316 | 1.521 | 0.988-2.342 | 0.057 | 1.509 | 1.042-2.186 | 0.029 |

Multivariate disease-specific and overall survival analyses according to the Cox proportional hazards regression model for 645 patients with endometrial cancer. Weekday of surgery was dichotomized as either early-week (Monday and Tuesday), or late-week (Wednesday through Friday).

CI = Confidence Interval, FIGO = International Federation of Gynecology and Obstetrics, HR = Hazard Ratio.

^1^Age of primary treatment and BMI evaluated as continuous variables.

^2^Histology evaluated in postoperatively acquired hysterectomy specimens
